# Longitudinal cohort study of discrepancies between prescribed and administered polypharmacy rates: implications for National Aged Care Quality Indicator Programs

**DOI:** 10.1136/bmjqs-2023-017042

**Published:** 2024-07-16

**Authors:** Nasir Wabe, Rachel Urwin, Karla Seaman, Johanna I Westbrook

**Affiliations:** 1 Australian Institute of Health Innovation, Faculty of Medicine and Health Sciences, Macquarie University, Sydney, New South Wales, Australia

**Keywords:** Nursing homes, Healthcare quality improvement, Health services research, Performance measures

## Abstract

**Background:**

Polypharmacy is frequently used as a quality indicator for older adults in Residential Aged Care Facilities (RACFs) and is measured using a range of definitions. The impact of data source choice on polypharmacy rates and the implications for monitoring and benchmarking remain unclear. We aimed to determine polypharmacy rates (≥9 concurrent medicines) by using prescribed and administered data under various scenarios, leveraging electronic data from 30 RACFs.

**Method:**

A longitudinal cohort study of 5662 residents in New South Wales, Australia. Both prescribed and administered polypharmacy rates were calculated biweekly from January 2019 to September 2022, providing 156 assessment times. 12 different polypharmacy rates were computed separately using prescribing and administration data and incorporating different combinations of items: *medicines and non-medicinal products*, *any medicines* and *regular medicines* across four scenarios: no, 1-week, 2-week and 4-week look-back periods. Generalised estimating equation models were employed to identify predictors of discrepancies between prescribed and administered polypharmacy.

**Results:**

Polypharmacy rates among residents ranged from 33.9% using data on administered *regular medicines* with no look-back period to 63.5% using prescribed *medicines and non-medicinal products* with a 4-week look-back period. At each assessment time, the differences between prescribed and administered polypharmacy rates were consistently more than 10.0%, 4.5%, 3.5% and 3.0%, respectively, with no, 1-week, 2-week and 4-week look-back periods. Diabetic residents faced over two times the likelihood of polypharmacy discrepancies compared with counterparts, while dementia residents consistently showed reduced likelihood across all analyses.

**Conclusion:**

We found notable discrepancies between polypharmacy rates for prescribed and administered medicines. We recommend a review of the guidance for calculating and interpreting polypharmacy for national quality indicator programmes to ensure consistent measurement and meaningful reporting.

**WHAT IS ALREADY KNOWN ON THIS TOPIC**

- Polypharmacy (the use of multiple medicines) is prevalent among older adults and is often used as a quality of care indicator in aged care settings, despite being poorly defined.

**WHAT THIS STUDY ADDS**

- Polypharmacy rates are highly variable when calculated using either prescribing or administration data, different timeframes for data review and the inclusion criteria for medicines.

**HOW THIS STUDY MIGHT AFFECT RESEARCH, PRACTICE OR POLICY**

- We recommend a more consistent approach to the measurement of polypharmacy to enable more meaningful reporting and appropriate quality of care benchmarking in aged care settings.

## Introduction

 Medication safety is a global public health priority.^1^ Poor medication management is particularly prominent in residential aged care facilities (RACFs, also known as care homes, and nursing homes), with almost all residents identified as facing at least one medicine-related issue during their stay.^2^ One-third of complaints to the Australian Royal Commission into Aged Care Quality and Safety were related to medicines, highlighting the need for close monitoring of the quality and safety of medication use.^3^ A range of initiatives and interventions were implemented as part of the national response,^4^ which included the establishment of a National Aged Care Mandatory Quality Indicator Program (NACMQIP) to monitor the performance of RACFs through the quarterly reporting of 11 quality indicators across key areas of care, including two indicators related to medication management; antipsychotic use and polypharmacy.^5 6^

Polypharmacy, or the concurrent use of multiple medicines,^7^ is prevalent in RACFs due to the number of medicines prescribed to manage acute and chronic health conditions of residents.^8^,^10^ The use of polypharmacy as a quality measure has been debated in Australia and internationally for several reasons.^11^ First, polypharmacy, while indicative of the disease burden, does not offer clinically meaningful information about the appropriateness of medicines use, requiring additional tools and further investigation for a comprehensive assessment.^12^,^15^ Second, there is no standard definition of polypharmacy, both in terms of number of medicines and the way it is measured,^9 16^ which can limit the value of polypharmacy comparisons across different studies and settings.^11 17 18^ Third, the lack of reliability of polypharmacy as a measure to assess differences in quality of care between aged care providers and/or facilities or to identify individuals at risk of harm from their medicines, limits its utility for benchmarking and quality improvement.^11 19^

Despite these limitations, several countries have adopted polypharmacy as a quality indicator for RACFs.^6 11 19 20^ In Australia, the polypharmacy national quality indicator is defined as the prescription of nine or more medicines to a care recipient, calculated through a review of the medication charts or administration records for each care recipient on one selected collection date every quarter.^6^ There is limited guidance about whether prescription data (reflecting the medicines that are prescribed) or administration data (the medicines that are actually administered or taken by residents) should be used in the calculation of polypharmacy. Furthermore, the time point at which polypharmacy data should be collected is not stipulated and is therefore chosen at the aged care providers’ discretion (eg, first day, last day, midpoint of quarter). In addition to these potential differences in polypharmacy calculation by providers, other factors also have the potential to influence the calculation of polypharmacy rates. These may include instances such as when dispensed medicines are not administered due to resident refusal or absence from the facility, or occasions when medicines are taken at a later time but the medication management system is not updated by staff. Additionally, the periodic administration of some medicines through injections or dermal patches (e.g., a vitamin B12 injection administered every 3 months) can also fall into this category. Understanding how data sources, time period selection and other factors may influence polypharmacy rates is critical at a national level to be able to interpret national indicators and for RACFs to successfully monitor and manage medicines use among residents.

To investigate the reliability of polypharmacy rates based on the current NACQIP guidelines, we calculated polypharmacy rates under various scenarios using routinely collected electronic medication data from 30 Australian RACFs managed by two aged care providers. The objectives of the study were to identify (1) any differences in polypharmacy rates calculated using prescription or administration data and (2) predictors of differences between prescribed and administered polypharmacy rates. Through this exploration, we aim to determine a more reliable definition of polypharmacy, which will carry significant implications for reporting guidelines within the National Aged Care Quality Indicator Program and for other countries seeking to use polypharmacy as a quality indicator.

## Methods

### Study design and setting

This retrospective longitudinal cohort study used electronic aged care medication data sourced from 30 RACFs, including 24 facilities from provider A and 6 from provider B in New South Wales, Australia. The study period was from 1 January 2019 to 30 September 2022. We structured this paper following the REporting of studies Conducted using Observational Routinely collected health Data statement.^21^

### Participants

The study participants consisted of individuals aged 65 years or older who were receiving care on or after 1 January 2019. We have incorporated all residents, regardless of permanent or respite status, in adherence to the stipulations of the NACMQIP. Interim care (temporary entry) residents were excluded from the study because of the short-term nature of their stays.

### Data source

We used routinely collected electronic data from aged care providers, linking two databases—resident profiles and electronic medication administration record (eMAR). The resident profile database contained demographic details (eg, age, gender, care provider) and information on each resident’s health status at the time of entry into RACF (eg, dementia status, diabetes). The eMAR contained details about the daily medicines and non-medicinal products administered to each resident, as well as those scheduled but not administered, as depicted in online supplemental figure. We employed the WHO’s Anatomical Therapeutic Classification (ATC) level five codes to identify the daily medicine profiles of residents. Each medicine was counted once per day, irrespective of the frequency or dosage of administration. The total count of unique medicine entries in the eMAR on any given day, covering both administered and non-administered medicines, represented *prescribed* medicines, whereas the count of administered entries solely indicated *administered* medicines.

### Polypharmacy definition

Polypharmacy was defined as the percentage of care residents who received nine or more medicines and or non-medicinal products as presented in table 1. We calculated three versions of polypharmacy rates for both prescribed and administered medicines, using: (1) *medicines and non-medicinal products (eg, antiseptics*), (2) *any medicines* (including short course and medicines taken as needed (PRN) but excluding supplements (eg, vitamins) and other non-medicinal products) and (3) *regular medicines* (excluding supplements, non-medicinal products, short-course medicines and PRN). In our study, regular medicines comprised drugs prescribed for chronic conditions. The term ‘regular’ medicines is consistent with the definition used in the NACMQIP.^6^ Short-term medicines in this study included dermatologicals (ATC code D), systemic anti-infectives (ATC code J), gynaecological anti-infectives and antiseptics (ATC code G01), anti-infective preparations for ophthalmological use (ATC code S01A), otological use (ATC code S02A), eye or ear use (ATC code S03A), intestinal anti-infectives (ATC code A07A), anti-infectives and antiseptics for local oral treatment (ATC code A01AB), combination medicines for the eradication of *Helicobacter pylori* (ATC code A02BD) and anti-infectives and antiseptics for local oral treatment (ATC code R02AB). Non-medicinal products include essential items for resident care that are not classified as medications but are tracked within the eMAR system. Examples include supplements (eg, minerals), medical devices such as insulin pumps and nebulizers, personal care products such as mouth care items, incontinence supplies and wound dressings, as well as therapeutic devices such as heating pads and physical therapy equipment.

**Table 1:** Medicines and non-medicinal products included in the calculation of polypharmacy rates

What was included in the polypharmacy calculation?	Medicines		Non-medicinal products
	Regular	Short course/PRN	Supplements and other products*
Medicines and non-medicinal products			
Any medicines†			
Regular medicines‡			

* E.g.For example, Aantiseptics, mouth care.

† Excluded supplements and non-medicinal products.

‡ Excluded supplements, non-medicinal products, short-course medicines, and PRN (pro re nata, medicines taken as needed).

The NACMQIP mandates reporting polypharmacy on a specific review date (which is selected by each provider) each quarter without a look-back period.^6^ In our study, in addition to calculating polypharmacy rates without a look-back period, we broadened our analysis by incorporating various look-back periods to evaluate differences in polypharmacy rates when comparing prescribed and administered medicines. Specifically, we assessed the status of polypharmacy on a biweekly basis between January 2019 and September 2022, resulting in a total of 233 138 biweekly polypharmacy values across 156 assessment time points. The following four polypharmacy assessment data points were considered in the current study: a biweekly review date without a look-back period, a biweekly review date with a 1-week look-back period, a biweekly review date with a 2-week look-back period and a biweekly review date with a 4-week look-back period. Polypharmacy reporting based on a single-day review or daily data may lead to rate variations across facilities, given the increased daily volatility in patterns. Introducing a look period helps aggregate data to weekly levels or beyond, potentially minimising fluctuations in polypharmacy rates across facilities. Polypharmacy rates, determined through a look-back period, assessed whether nine or more unique medications were prescribed or administered over a specified duration.

### Statistical analysis

We reported descriptive statistics including frequency with percentage (%) for categorical variables and median with IQR for continuous variables. We employed generalised estimating equations (GEEs) to identify the longitudinal predictors of discrepancies between prescribed and administered polypharmacy rates. We focused on the polypharmacy rates calculated using regular medicines for this analysis. At each assessment point, we assessed the presence of discrepancies between prescribed and administered polypharmacy medicines (Y/N), resulting in 233 138 biweekly binary outcome measures of discrepancy. In the GEE model, we employed a binomial distribution with a logit link function and robust SE to address the panel nature of the data. The potential predictors considered in the analysis included baseline demographic variables (”age, sex, an indicator of whether the resident was new or existing at the start of the study and providers (A or B) as well as baseline health conditions”, as presented in table 2 (eg, dementia, Parkinson’s disease). The strength of association was estimated using the OR with 95% CI. All p values were two tailed, and statistical significance was set at p<0.05. The analysis was carried out using Stata V.18 (StataCorp LP, College Station, Texas, USA).

**Table 2:** Baseline participant characteristics (n=5662)

Variable	N (%)
Female	3673 (64.9)
Age in years, median (IQR)	86 (80–90)
Age group	
<80	1276 (22.5)
80–85	1494 (26.4)
86–89	1258 (22.2)
≥90	1634 (28.9)
Provider	
A	4269 (75.4)
B	1393 (24.6)
Cohort	
Entered the facility on or after January 2019	3029 (53.5)
Entered the facility before January 2019	2633 (46.5)
Heath status	
Any circulatory conditions	5010 (88.5)
Cerebrovascular accident	1420 (25.1)
Any endocrine conditions	2193 (38.7)
Diabetes	1588 (28.1)
Thyroid	606 (10.7)
Chronic respiratory disease	1043 (18.4)
Cancer	1655 (29.2)
Parkinson’s disease	445 (7.9)
Peptic ulcer/gastro-oesophageal reflux disease	1758 (31.1)
Renal disease	1085 (19.2)
Dementia	2919 (51.6)
Osteoporosis	1627 (28.7)
Arthritis	3239 (57.2)
Gout	561 (9.9)
Fracture	1930 (34.1)
Hearing impairment	1235 (21.8)
Depression, mood and affective disorders	2542 (44.9)
Anxiety and stress-related disorders	1699 (30)
Visual impairment	947 (16.7)
Medicines (ATC level 1)	
Alimentary tract and metabolism	2733 (48.3)
Anti-infectives for systemic use	837 (14.8)
Antineoplastic and immunomodulating agents	152 (2.7)
Antiparasitic products, insecticides and repellents	25 (0.4)
Blood and blood-forming organs	1859 (32.8)
Cardiovascular system	2386 (42.1)
Dermatologicals	599 (10.6)
Genitourinary system and sex hormones	456 (8.1)
Musculoskeletal system	612 (10.8)
Nervous system	2461 (43.5)
Respiratory system	634 (11.2)
Sensory organs	810 (14.3)
Systemic hormonal preparation	708 (12.5)

ATC, Anatomical Therapeutic Classification

## Results

### Participants

A total of 5662 participants met the inclusion criteria. Participants had a median age of 86 years (IQR 81–91), with 64.9% being women and 51.6% having a dementia diagnosis at baseline. Over half of the residents (53.5%, n=3029) entered the facility on or after January 2019 (table 2).

### Prescribed and administered polypharmacy rates

There was a total of 233 138 biweekly polypharmacy assessment data points during the study period. In table 3, prescribed and administered polypharmacy rates are presented for each look-back period across all 233 138 assessments. Polypharmacy rates ranged from 33.9% using administered regular medicines with no look-back period to 63.5% using prescribed medicines and no-medicinal products with 4-week look-back period. Regardless of the components included in the polypharmacy calculation, there was consistently over a 10.0%, 4.5%, 3.5% and 3.0% difference between prescribed and administered polypharmacy rates with no, 1-week, 2-week and 4-week look-back periods, respectively. For example, when polypharmacy was calculated using regular medicines (excluding short courses and PRN), the prescribed and administered polypharmacy rates were 44.2% and 33.9%, respectively, resulting in a difference of 10.3%.

**Table 3:** Percentage of residents with polypharmacy derived from 233 138 biweekly assessment data points between January 2019 to September 2022

N (%)	What was included in the polypharmacy calculation?		
	Medicines and non-medicinal products	Any medicines	Regular medicines
No look-back period			
Prescribed	129 445 (55.5)	116 361 (49.9)	102 950 (44.2)
Administered	103 422 (44.4)	91 301 (39.2)	79 034 (33.9)
Difference	26 023 (11.1)	25 060 (10.7)	23 916 (10.3)
1-week look-back period			
Prescribed	140 120 (60.1)	127 171 (54.6)	111 216 (47.7)
Administered	129 213 (55.4)	115 847 (49.7)	100 171 (43.0)
Difference	10 907 (4.7)	11 324 (4.9)	11 045 (4.7)
2-week look-back period			
Prescribed	143 371 (61.5)	130 671 (56.1)	113 020 (48.5)
Administered	135 168 (58.0)	121 871 (52.3)	104 517 (44.8)
Difference	8203 (3.5)	8800 (3.8)	8503 (3.7)
4-week look-back period			
Prescribed	148 124 (63.5)	135 684 (58.2)	115 963 (49.7)
Administered	141 631 (60.8)	128 498 (55.1)	109 016 (46.7)
Difference	6493 (2.7)	7186 (3.1)	6947 (3.0)

Both prescribed and administered polypharmacy rates increased with increasing look-back weeks, but the differences between the two rates narrowed, although a difference of approximately 3.0% was still observed even with a 4-week look-back period. For instance, using *medicines only* in polypharmacy calculations, when moving from no to 4-week look-back periods, the rates increased from 49.9% to 58.2% (prescribed rate) and from 39.2% to 55.15% (administered rate) (table 3).

### Prescribed and administered polypharmacy rates over time (January 2019 to September 2022)

**Figure 1:**
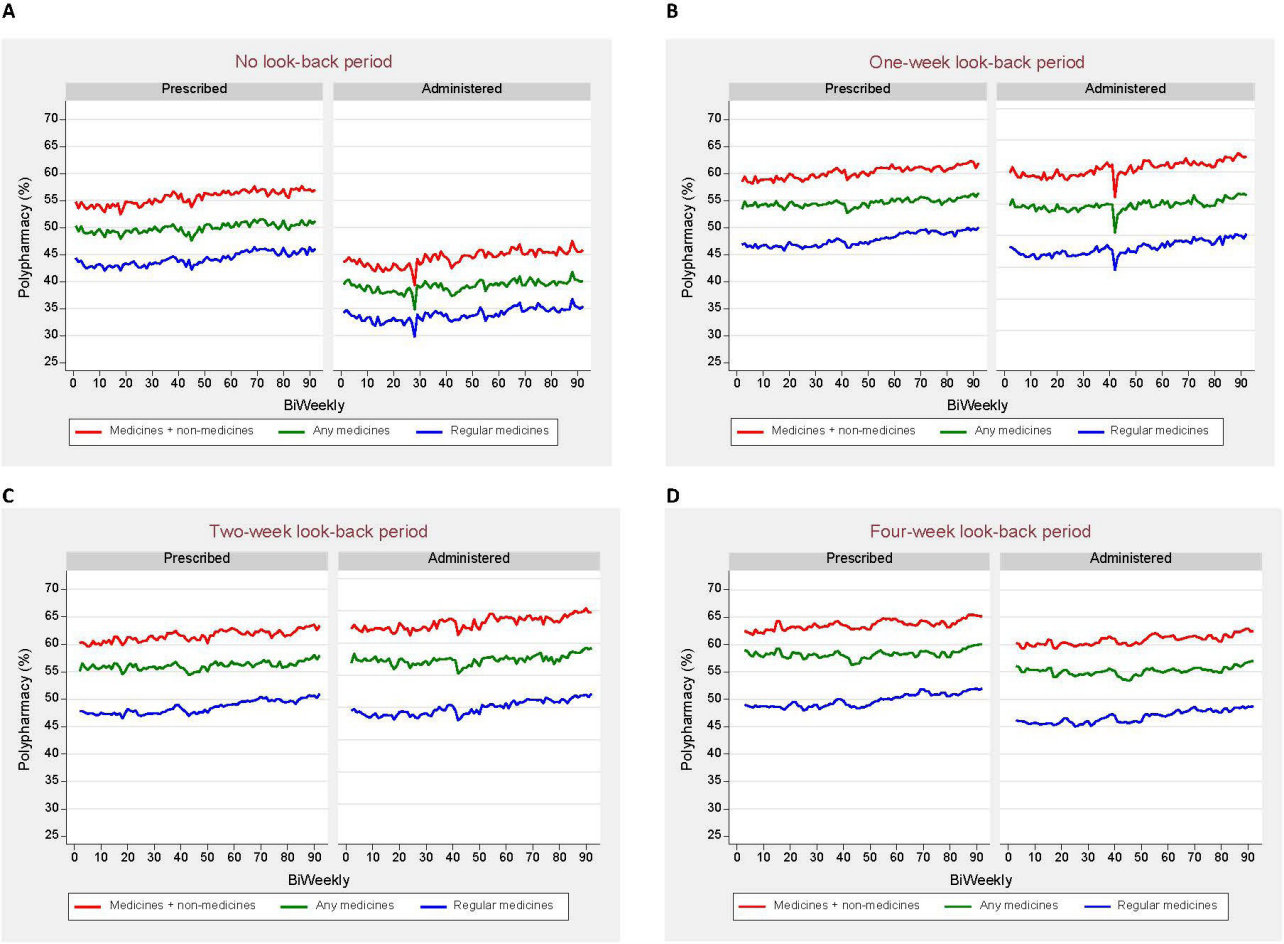
Trends in prescribed and administered polypharmacy rates by look-back period.

Figure 1 illustrates trends in polypharmacy rates for the four look-back periods. No significant changes or fluctuations over time were observed for all look-back periods, except for some variations in administered polypharmacy rates in 2020. Regardless of the components included in the polypharmacy calculation, differences of >10.0% between prescribed and administered polypharmacy rates were consistently maintained over time. That means at any given assessment time, the differences between the two rates were approximately 10% (figure 1A). Similarly, differences of approximately 4.5%, 3.5% and 3.0% were sustained over time for 1-week, 2-week and 4-week look-back periods, respectively (figure 1B–D).

### Predictors of discrepancies between prescribed and administered polypharmacy rates

Table 4 presents the results of GEE models identifying factors associated with the discrepancy between prescribed and administered rates using polypharmacy rates calculated using regular medicines. Age is inversely associated with the likelihood of experiencing a discrepancy between prescribed and administered polypharmacy rates for no look-back period analysis. In other words, increasing age was associated with decreased discrepancy—for example, residents aged <80 years were 1.39 times more likely to experience a discrepancy versus those aged ≥90 years (OR 1.39, 95% CI 1.19 to 1.62, p<0.001).

**Table 4:** GEE models showing predictors of discrepancies between prescribed and administered polypharmacy rates

	No look-back	One-week look-back	Two-week look-back	Four-week look-back
	OR (95% CI)	OR (95% CI)	OR (95% CI)	OR (95% CI)
Demographics				
Male versus female	1.11 (0.98 to 1.25)	1.14 (0.97 to 1.34)	1.16 (0.98 to 1.37)	1.13 (0.95 to 1.36)
Age group (Ref≥90 years old)				
<80	**1.39(1.19to1.62)**	1.2 (0.98 to 1.46)	1.15 (0.93 to 1.41)	1.12 (0.90 to 1.410
80–85	**1.39(1.20to1.60)**	**1.25(1.03to1.51)**	1.15 (0.94 to 1.41)	1.09 (0.88 to 1.35)
86–89	**1.30(1.13to1.51)**	1.17 (0.97 to 1.43)	1.11 (0.90 to 1.36)	1.08 (0.86 to 1.35)
Provider A versus provider B	0.92 (0.82 to 1.04)	0.95 (0.82 to 1.11)	0.90 (0.77 to 1.06)	0.95 (0.79 to 1.14)
New versus existing cohort	1.04 (0.94 to 1.16)	0.99 (0.86 to 1.14)	0.97 (0.83 to 1.12)	1.03 (0.88 to 1.22)
Health status				
Cerebrovascular accident	1.11 (0.99 to 1.24)	1.09 (0.94 to 1.27)	1.11 (0.95 to 1.30)	1.07 (0.89 to 1.28)
Diabetes	**2.63(2.36to2.94)**	**2.60(2.25to3.01)**	**2.41(2.07to2.81)**	**2.11(1.78to2.50)**
Thyroid	**1.28(1.10to1.50)**	1.14 (0.90 to 1.44)	1.10 (0.86 to 1.42)	1.21 (0.93 to 1.58)
Chronic respiratory disease	**1.30(1.15to1.46)**	**1.19(1.01to1.40)**	1.07 (0.90 to 1.28)	0.99 (0.81 to 1.20)
Neoplasms	1.04 (0.92 to 1.17)	1.13 (0.97 to 1.31)	1.14 (0.98 to 1.33)	1.16 (0.98 to 1.37)
Parkinson’s disease	**1.38(1.16to1.65)**	1.13 (0.89 to 1.45)	1.12 (0.87 to 1.45)	1.08 (0.81 to 1.43)
Peptic ulcer/gastro-oesophageal reflux disease	**1.24(1.12to1.38)**	**1.17(1.02to1.35)**	1.11 (0.96 to 1.29)	1.04 (0.88 to 1.22)
Renal disease	**1.36(1.20to1.53)**	**1.32(1.13to1.55)**	**1.27(1.08to1.50)**	1.11 (0.92 to 1.33)
Dementia	**0.60(0.54to0.67)**	**0.68(0.59to0.78)**	**0.74(0.64to0.86)**	**0.81(0.69to0.95)**
Osteoporosis	1.08 (0.96 to 1.21)	1.17 (1.00 to 1.37)	**1.21(1.02to1.43)**	**1.27(1.06to1.52)**
Arthritis	1.07 (0.96 to 1.20)	1.02 (0.88 to 1.18)	0.99 (0.85 to 1.16)	0.98 (0.83 to 1.16)
Gout	**1.27(1.08to1.49)**	1.23 (0.99 to 1.53)	1.21 (0.96 to 1.53)	1.22 (0.94 to 1.59)
Fracture	1.06 (0.95 to 1.19)	1.04 (0.89 to 1.21)	1.09 (0.93 to 1.28)	1.17 (0.99 to 1.39)
Hearing impairment	0.94 (0.831.07)	**0.77(0.65to0.92)**	**0.77(0.64to0.92)**	**0.75(0.61to0.91)**
Depression, mood and affective disorders	**1.16(1.04to1.30)**	1.02 (0.88 to 1.18)	0.97 (0.83 to 1.13)	0.95 (0.80 to 1.12)
Anxiety and stress-related disorders	**1.19(1.06to1.33)**	1.11 (0.95 to 1.3)	1.07 (0.90 to 1.26)	1.00 (0.83 to 1.21)
Visual impairment	1.02 (0.89 to 1.16)	1.09 (0.91 to 1.31)	1.10 (0.92 to 1.33)	1.15 (0.94 to 1.42)

P<0.05 was indicated in bold.

GEE, generalised estimating equations

Residents with diabetes exhibited a consistently greater likelihood of discrepancy between prescribed and administered polypharmacy rates, more than two times as high as their counterparts, regardless of the chosen look-back period. For instance, in the no look-back analysis, residents with diabetes had a 2.63 times greater likelihood of experiencing a discrepancy compared with their counterparts (OR 2.63, 95% CI 2.36 to 2.94, p<0.001). Examples of other health conditions associated with a greater likelihood of discrepancy in at least one of the analyses included chronic respiratory disease, renal disease and osteoporosis. Interestingly, however, having dementia was consistently linked with a decreased likelihood of discrepancy across all four look-back analyses (eg, OR 0.81, 95% CI 0.69 to 0.95, p=0.010 for 4-week look-back analysis) (table 4).

## Discussion

Our findings demonstrate that rates of polypharmacy in RACFs are highly variable, depending on the use of prescribing versus administration data, the timeframe for data review and the inclusion criteria for medicines. Polypharmacy rates calculated using NACMQIP inclusion criteria (ie, regular medicines excluding supplements, non-medicinal products, short-course medicines and PRN) and data records for prescribed medicines compared with administered medicines indicated a 10% difference in polypharmacy rates (44.2% prescribed vs 33.9% administered). These discrepancies declined when the 1-day medicines review included a retrospective data collection period and were reduced to a 3% difference when incorporating a 4-week look-back period. Residents with dementia had a reduced likelihood of polypharmacy discrepancies, while residents with diabetes had two times the likelihood of discrepancies in prescribed and administered polypharmacy compared with those without.

The present study underscores the pivotal role of data sources in polypharmacy calculation. Using eMARs integrated into routine resident care, our study identified a 10% difference between prescribed and administered polypharmacy rates based on a single review date. Findings from a systematic review reveal a diverse range of data sources for polypharmacy calculation.^9^ In a comprehensive analysis of 44 polypharmacy studies in long-term care facilities, Jokanovic *et al*^9^ identified various sources, with medication charts or medical records being the primary source in 66% of the studies. Other sources, including drug registries, minimum data sets, resident interviews and pharmacist-led medication reviews, were also used. While most studies in the review included prescribed medicines in polypharmacy calculations, five studies noted medication administration records as the data source without specifying whether the calculations were based on administered or prescribed medicines.^9^ The limited use of medication administration records reported in the literature could be attributed to the low implementation of eMAR in RACFs.^22^ The use of electronic medication management systems can reduce medication safety risks and decrease the administrative burden for prescribers, pharmacists and staff,^23^,^25^ although the uptake has been slow.^22 26^ While all Australian RACFs do not currently have electronic medication management systems, the Australian government has recently provided financial support to residential aged care providers to adopt Electronic National Residential Medication Charts software,^27^ which will provide opportunities to standardise polypharmacy calculation in the future.

Polypharmacy rates for prescribed and administered medicines increased when the medication review period was extended from a single day to include look-back periods of 1, 2 or 4 weeks. The discrepancy between prescribed and administered polypharmacy rates decreased as the look-back period increased (3.0% difference with 4-week look back, compared with 10.3% difference with no look back), highlighting the dynamic daily medication needs of residents,^28^ the extent of missed or delayed medicines^29 30^ or the periodic (ie, not daily) administration of some medicines such as transdermal patches.^31^ The adoption of a single day review for NACMQIP polypharmacy reporting likely results in an underestimate of polypharmacy experienced in Australian RACFs and contrasts with previously reported definitions of polypharmacy that specify longer look-back periods,^10^ such as 7 days,^19^ 3 months^32^ or 1 year.^33^

By including a range of definitions of polypharmacy in our analysis, we were able to explore the actual day-to-day care and support needs of residents as well as the potential medication burden and administration complexity for RACF staff. For example, counting all medicines (including short-term and PRNs) and non-medicinal administrations increased daily rates of prescribed polypharmacy to 55% and administered polypharmacy to 44%. The management of PRN medicines and their contribution to the medication burden in aged care settings is influenced by a range of resident characteristics and human factors.^34 35^ A systematic review of PRN use in nursing homes estimated that 48.4%–97.4% of residents were prescribed at least 1 PRN medicine^36^ and a recent study estimated that, over a 7-day period, more than one-third of residents received (ie, were administered) a prescribed PRN medicine^37^ and this did not change greatly over the course of 1 year. The number of medicines and the medication regimen complexity contribute to a resident’s medication burden,^28 38^ and may increase medication administration errors,^39 40^ lead to reduced adherence to medicines and increase the risk of hospitalisation, hospital readmissions and mortality among RACF residents.^41^ Given the potential of patient harm from frequent or inappropriate administration of PRN medicines,^42^ exactly what medicines should be included in the polypharmacy count requires further consideration. Furthermore, an under-reporting of polypharmacy could occur if, for example, a resident has been prescribed an analgesic such as paracetamol as a PRN medicine, which is then administered every day as a PRN rather than as a regularly scheduled daily medicine.

From a quality measurement perspective, identifying factors that predict discrepancies between prescribed and administered medications helps us recognise which populations are more vulnerable to deviations from prescribed medications. When looking only at regular medicines and taking into account demographic and clinical characteristics, the discrepancies in prescribed and administered polypharmacy were reduced with age and dementia status. In community settings, older adults with dementia are reported to have low levels of medication adherence,^43^ with caregivers playing a critical role in therapeutic management.^44^ There are limited comparative studies in aged care settings, although residents are often reliant on staff for medication administration^4^ and studies report that nursing home residents with dementia often spend large proportions of their day in their rooms, engaged in no or very few activities,^45^,^47^ which could explain our findings of relative concordance between prescribed and administered medicines among these individuals. Conversely, the discrepancies in prescribed and administered polypharmacy increased with health conditions such as Parkinson disease and diabetes. While the reasons for these deviations need to be explored in future studies, our findings imply potential non-adherence issues within the facilities for this subpopulation. Both Parkinson disease and diabetes often require complex medication regimens.^48^,^50^ For example, residents with diabetes may have medicines adjusted on a daily basis, are more likely to receive PRN medicines to support these adjustments and their medicines may not be managed according to current guidelines.^51^ The current NACMQIP polypharmacy indicator does not capture these complexities and a consideration of including PRNs or certain PRNs would be appropriate.

### Implications for practice and policy

Our findings provide several important considerations for the way the polypharmacy indicator is calculated by Australian RACFs for the NACMQIP. A more consistent approach to the polypharmacy calculation is needed. Ambiguities in the indicator definition, such as which medicines should be included in the nine or more count, whether medicines that are administered provide a more accurate reflection of polypharmacy than prescribed medicines, and the use of a single arbitrary review date, should be resolved for the national quality indicator programme to provide meaningful feedback to providers, residents and the broader community. Moreover, our study highlights the challenges associated with estimating polypharmacy based on only a numerical definition (in this case nine or more medicines) and does not explore ongoing concerns about the clinical appropriateness of the use of multiple medicines for an individual in a RACF.^11^ Our results are equally relevant to quality indicator programmes in other countries, many of which include polypharmacy. Our findings form the basis for future studies to demonstrate the evidence that a polypharmacy indicator will help identify residents at higher risk of harm from their medicines and lead to improved medication management in aged care settings.^52^

Our study highlighted significant variability in polypharmacy rates, depending on different scenarios: the type of data used (prescribed vs administered), the inclusion of various substances (eg, medicinal products, supplements) and the data collection window (look-back periods), with rates ranging from 33.9% to 63.5%. This prompts the question of what constitutes the preferred polypharmacy definition for use in national quality indicator programmes. While multiple considerations are necessary, such as ease of access to administration data, using administered data with a specific look-back period (eg, 2 weeks) may be preferable to the current practice of single-day data collection using prescribed data.^6^ A 4-week look-back period may be less ideal because some medications may be discontinued within that time frame. However, redefining the national polypharmacy quality indicator should involve consultation with consumers, aged care providers and policymakers. It is imperative to balance an indicator that has meaningful implications with one that can be readily calculated. An indicator generated in this way will enable more practical applications and provide accurate benchmarking. Another important consideration is what should be included in the polypharmacy calculation. The current Australian definition includes only regular medicines. Given the high prevalence of supplement usage in RACFs, which may pose a significant burden and raise quality use issues, further research is needed to determine whether supplements should be included in the polypharmacy definition.

### Strengths and limitations of the study

This pioneering study represents the first effort to compare polypharmacy rates by considering both prescribed and administered medicines. The incorporation of administered medication data enhances the precision of documenting the medicines received by each resident. Additional strengths of our research include a large sample size, an extended follow-up duration and a multicentre approach involving 30 facilities. However, while the innovative methods employed to examine this issue are applicable regardless of geographic area, a notable limitation is the concentration of data in our study from RACFs within the Sydney metropolitan area, potentially limiting the generalisability of our findings to RACFs nationwide. There may be variations in prescribing practices, medicines use and polypharmacy rates in rural areas and across different states and territories in Australia. While our analyses considered several factors influencing the polypharmacy rate during the study period, it is important to acknowledge the potential influence of other unexamined factors in our study. For instance, the pandemic disrupted usual prescribing patterns,^53^ and the introduction of the national quality indicator programme in Australia^6^ may have impacted prescribing behaviours. Nonetheless, by calculating the prescribed versus administered polypharmacy rates at the same point in time, our analyses ensured consistency in accounting for these influencing factors across all calculations. Future investigations should prioritise a more comprehensive representation across diverse geographical settings to enhance the broader applicability of our findings within the Australian context and beyond.

## Conclusion

We observed notable discrepancies between prescribed and administered polypharmacy rates, underscoring the considerable variability in polypharmacy rates within RACFs. This variability is influenced by factors such as the choice between prescribing and administration data, the timeframe for data review and the inclusion criteria for medicines in polypharmacy calculation. We recommend that the government with national key bodies and consumers review the polypharmacy national indicator to enable more meaningful and consistent polypharmacy measurement and reporting to the NACMQIP across Australian RACFs. Likewise, countries using polypharmacy as a quality indicator should consider the implication of these findings for their programmes.

## supplementary material

10.1136/bmjqs-2023-017042 online supplemental file 1

## Data Availability

Data are available on reasonable request.
