# Supplementary material for: Longitudinal cohort study of discrepancies between prescribed and administered polypharmacy rates: implications for National Aged Care Quality Indicator Programs
Source: BMJ Qual Saf. 2024 Jul 16;33(12):e017042. doi: 10.1136/bmjqs-2023-017042 (PMC11671870; doi:10.1136/bmjqs-2023-017042)
Supplement: online supplemental file 1 [file bmjqs-33-12-s001.pdf]

id	Meds_Admin_Dateonly	Administered	Level_5_name
1	05jan2019	No	PREDNISONE
1	05jan2019	No	BISOPROLOL
1	05jan2019	No	ACETYLSALICYLIC ACID
1	05jan2019	No	DOMPERIDONE
1	05jan2019	No	PERINDOPRIL
1	05jan2019	No	COLECALCIFEROL
1	05jan2019	No	GLYCERYL TRINITRATE
1	05jan2019	No	FUROSEMIDE
1	05jan2019	No	EZETIMIBE
1	05jan2019	Yes	MAGNESIUM
1	05jan2019	Yes	PARACETAMOL
1	05jan2019	Yes	MOXONIDINE
1	05jan2019	Yes	SIMVASTATIN

Supplemental Figure: Example of medicine profile of one resident in the eMAR indicating prescribed and administered medicine on 05 Jan 2019.
